# Readmission Prediction in TAVI: Optimizing the Optimal Procedure∗

**DOI:** 10.1016/j.jacadv.2022.100073

**Published:** 2022-08-26

**Authors:** Benjamin Hibbert, Omar Abdel-Razek

**Affiliations:** Division of Cardiology, Department of Medicine, University of Ottawa Heart Institute, Ottawa, Ontario, Canada

**Keywords:** readmission, structural heart, TAVI, TAVR, transcatheter aortic valve implantation, transcatheter aortic valve replacement

**Figure:**
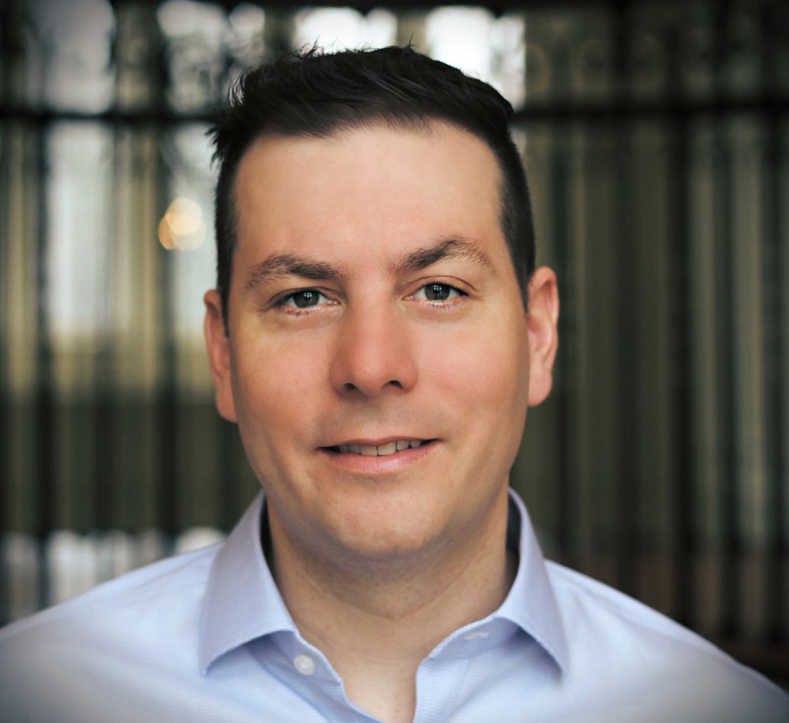

Transcatheter interventions continue to evolve improving the care of patients with a number of cardiac conditions.^1^, ^2^, ^3^ In particular, structural valve procedures are revolutionizing the care of patients with valvular heart disease, with randomized trial data to inform decisions regarding timing and type of intervention. The design and development of many of the device-based therapies follow a common trajectory. Beginning as complex team-based procedures requiring general anesthesia and multiple operators, they evolve into a more simplified model with single operators, conscious sedation only, rapid mobilization, and early discharge. Transcatheter aortic valve implantation (TAVI) has become a prime example of this type of development in the structural heart space. TAVI, once a complex procedure only for patients with prohibitively high surgical risk, is now a commonly performed across a wide risk spectrum of patients.

As procedural optimization has progressed, TAVI outcomes focus has shifted from procedural techniques to enhancing outcomes through understanding the patient-related and care pathway factors that predict poor outcome. Despite marked improvements in immediate outcomes, readmission is still common following TAVI.^4^ Given the high cost and frequency, readmission represents one of the few areas with significant opportunity for improvement in post-TAVI outcomes.

In this issue of *JACC: Advances*, Sulaiman et al^5^ use a machine learning approach and a contemporary large administrative data set to identify risk factors for readmission after TAVI. In their cohort, the 30- and 90-day readmission rates were 12.4% and 21.5%, respectively. Using this approach, the authors developed a risk score, which also had good performance in an external validation cohort. Similar to other work in this field, Sulaiman et al identified a number of nonmodifiable patient-related factors that predicted readmission after TAVI. The 5 main predictors of readmission included length of stay at the time of the index procedure, frailty score, total discharge diagnoses, acute kidney injury, and Elixhauser score. Compared with other models, the current study offers a simplified method to identify high-risk patients using readily available clinical data. However, with the exception of acute kidney injury (which is best predicted by baseline renal function), the predictors reflect patient selection rather than delivery of care.^6^ Furthermore, the cohort of at-risk patients is large and may represent as much as 20% of a contemporary TAVI cohort. Thus, although TAVI is feasible and can be offered to patients with high comorbid burdens, the ability to change readmission risk may be more difficult.

Ultimately, to optimize TAVI outcomes and minimize readmissions, a predictive model only remains useful if it is possible to modify the risks. One can envision improving TAVI readmission rates by identifying high-risk patients early, providing more timely postdischarge follow-up, and targeting this group for interventions. If these can be initiated, demonstrating a reduction in readmission may follow. TAVI truly is an optimal procedure, technically feasible with minimal risks and suitable for many patients. However, readmissions remain common. Can we optimize such an optimal procedure or is our ability to reduce readmissions limited by the high comorbid burden of the patients who are offered the procedure?

## Funding support and author disclosures

The authors have reported that they have no relationships relevant to the contents of this paper to disclose.
